# Rotigotine protects against oxidized low-density lipoprotein(ox-LDL)-induced damages in human umbilical vein endothelial cells(HUVECs)

**DOI:** 10.1080/21655979.2021.2000224

**Published:** 2021-12-03

**Authors:** Hui Kang, Hui Yu, Jingxiu Fan, Ge Cao

**Affiliations:** Department of Cardiovascular Surgery, West China Hospital of Sichuan University, Chengdu, China

**Keywords:** Cardiovascular diseases, Rotigotine, ox-LDL, HUVECs, NF-κB

## Abstract

Rotigotine is a non-ergoline dopamine agonist that has been licensed for the treatment of Parkinson’s disease. Cardiovascular diseases are the world’s leading cause of death. Ox-LDL- induced endothelial damages are involved in the initiation and progression of cardiovascular diseases. In this study, we assessed the beneficial properties of Rotigotine on ox-LDL-induced insults to HUVECs to highlight its potential use in the treatment of cardiovascular diseases. Our findings show that Rotigotine suppresses the expressions of low-density lipoprotein receptor (LDL-R), proprotein convertase subtilisin/kexin type 9 (PCSK-9), and sterol regulatory element-binding protein (SREBP-2). It also inhibits ox-LDL-induced cholesterol accumulation in endothelial cells (ECs), improves U937 monocytes adhesion, and decreases the representation of NADPH oxidase (NOX-4) and generation of reactive oxygen species (ROS) in endothelial cells (ECs). Furthermore, Rotigotine inhibited the expressions of both vascular cellular adhesion molecule-1 (VCAM-1) and intercellular adhesion molecule-1 (ICAM-1) in HUVECs and had anti-inflammatory efficacy in ox-LDL-induced cells by inhibiting the expressions of pro-inflammatory cytokines. Notably, Rotigotine inhibits the activation of nuclear factor-kappaB (NF-κB) by preventing nuclear translocation of NF-κB p65 and reducing the luciferase activity of NF-κB reporter. We, therefore, conclude that these effects of Rotigotine on HUVECs suggest that it may play a therapeutic role in cardiovascular diseases.

## 1. Introduction

Acute occurrences such as heart attacks and strokes occur when obstructions limit blood flow from the heart or the brain. The most common reason for this is atherosclerosis (AS), which is the accumulation of cholesterol, fats, and other substances on the inner and outer walls of arteries. This buildup is called plaque, and it can cause arteries to narrow [1,2]. Due to the vascular problems that ensue, AS is a prominent cause of death and is classified as an inflammatory disease constantly recurring on the arterial walls [3–5]. Several different cell types are involved in its etiology, including endothelial cells (ECs), monocytes, and smooth muscle cells (SMCs). The proliferation of smooth muscle cells leads to the formation of mature lesions but damages the artery walls’ endothelial cells in the first stage. Hypertension, cigarette smoking, immune injury, hypercholesterolemia, and diabetes are some of the main triggers of this injury to these cells [6]. All blood vessels are lined with ECs, which are key mediators of inflammatory responses. They are frequently activated in the setting of AS via lipid buildup in the vessel wall and inflammatory mediator exposure. In turn, inflammatory cells are recruited and maintained, expanding atherosclerotic plaque [7,8].

The malfunction of various pathways contributes to the development of AS, however, oxidized low-density lipoprotein (ox-LDL), besides eliciting an oxidative stress response and impairing cells, is thought to promote endothelial dysfunction and accelerate the proliferation of cells involved in the etiology of AS, such as SMCs, monocytes, and macrophages [9]. Steinberg and others have argued that an early stage of AS is the oxidation of LDL, eventually leading to atherogenesis [10,11]. This LDL oxidation in the vessel wall follows the generation of ROS by endothelial cells [12]. Statins are widely used for the treatment of hyperlipidemia, however, even after well-tolerated statin therapy, an obvious residual cardiovascular risk remains in many patients. To decrease LDL-C, a new pharmacologically therapeutic target, PCSK9 has been examined. It enhances low-density lipoprotein receptor (LDL-R) degradation and prevents its recirculation to the cell surface, thereby reducing LDL intake from circulation. Furthermore, for patients requiring additional reduction of LDL-C, such as in atherosclerotic cardiovascular disease or familial hypercholesterolemia, PCSK9 inhibitors have been approved [13]. Another key cholesterol regulator is sterol-regulatory element-binding protein (SREBP)-2, which could activate LDL-R, leading to enhanced cholesterol uptake and biosynthesis [14,15]. In one experiment to determine the relationship between AS and serum cholesterol, the interrelationship between the increase in serum cholesterol (both free and total) and AS was found to be statistically insignificant [16]. The macrophages in plaque release a number of cytokines, including interleukin-8 (IL-8) [17] and tumor necrosis factor-α (TNF-α) [18]. They also produce ROS [19] to induce inflammation. The formation, development, and destabilization of atherosclerotic plaques are directly affected by these molecules, and they cause further recruitment of monocytes in the arteries by activating adhesion molecules on ECs [20]. By increasing inflammatory cell recruitment and causing endothelial cell dysfunction, ROS induce vascular diseases [21].

In ECs, ox-LDL causes endothelial adhesion genes implicated in atherogenesis and endothelial dysfunction such as ICAM-1, monocyte chemoattractant protein-1 (MCP-1), and VCAM-1 to be expressed [22,23]. Although macrophages normally express few receptors for normal LDL, they can take up ox-LDL by ways of scavenger receptors. In a study designed to determine the potential role of ox-LDL in the recruitment of macrophages to vessel walls, it was found that in addition to this, ox-LDL also stimulated the adhesion of U937 cells to cultured ECs and initiated their aggregation with monocytes [24]. The presence of activated nuclear factor-κB (NF-κB) suggests the involvement of the transcription factor in AS [25]. The role of NF-κB in the stimulation of ECs has yielded conflicting results over the years. Compared to Takahara et al. [26] and Rajavashisth et al. [27], who found enhanced NF-κB activity, Ares et al. (28) reported suppression of NF-κB activation upon treatment of ECs with ox-LDL.

**Figure 1.**
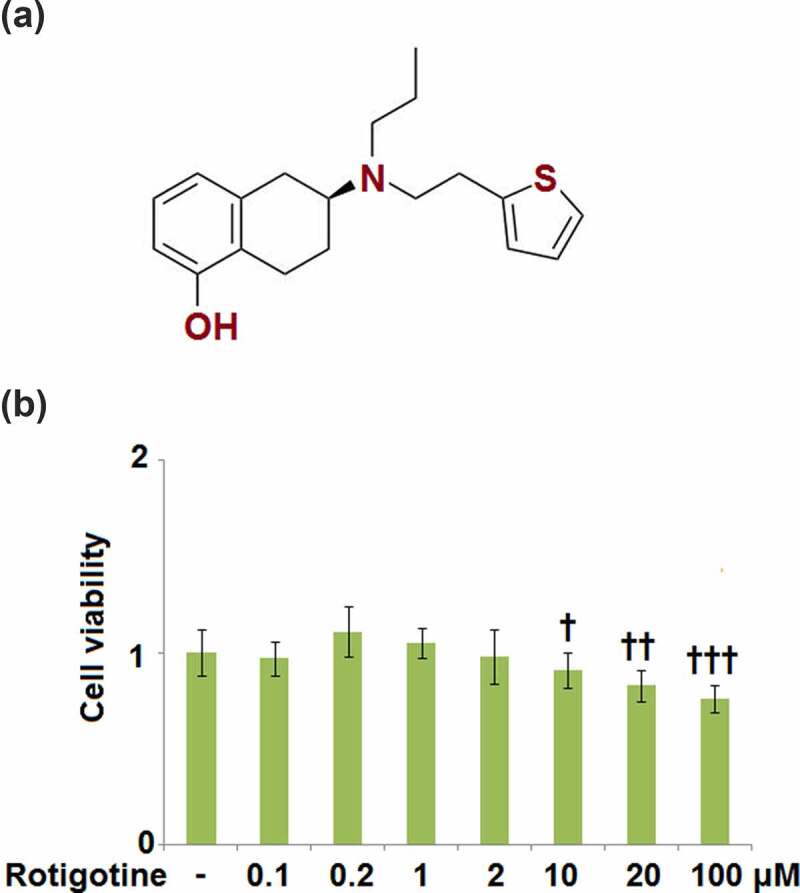
Cytotoxicity of Rotigotine in HUVECs. HUVECs were stimulated with 0.1, 0.2, 1, 2, 10, 20, and 100 μM Rotigotine for 24 hours. (a). Molecular structure of Rotigotine; (b). Cell viability (†, ††, †††, P < 0.01, 0.001, 0.0001 vs. vehicle group, N = 5–6)

Rotigotine (Neupro) (Figure 1(a)), a non-ergoline dopamine (DA) agonist, has been licensed for the treatment of Parkinson’s disease (PD) [28]. Rotigotine acts on all 5 DA receptors (D1-D5) but has displayed a higher affinity for D1, D2, and D3 receptors [29]. In one experiment, mice were treated using MPTP (1-methyl-4-phenyl-1,2,3,6-tetrahydropyridine), and the result was neuroinflammation and neurodegeneration. The Parkinsonism score was largely improved by the administration of Rotigotine, which also prevented microglial cell activation and the production of neuroinflammatory cytokines, and protected dopaminergic neurons with antioxidants. In the current study, we examined the beneficial effects of Rotigotine against ox-LDL- induced damage to human umbilical vein endothelial cells (HUVECs) to highlight a potential use of Rotigotine in the treatment of cardiovascular diseases.

## 2. Materials and methods

### 2.1. Cell culture and treatment

HUVECs were procured from ATCC and used in this investigation. These cells were cultured and grown in 2% serum endothelial growth media (EGM2) (Lonza, Switzerland) supplied with FBS (10%, v/v) and antibiotics at 37°C in a humidified 5% CO_2_ incubator. Cells were exposed to ox-LDL (100 µg/ml) (Sigma-Aldrich, USA) in the inclusion or exclusion 1 and 2 μM Rotigotine (Topscience, Shanghai, China) for another 24 hours [23].

### 2.2. 3-(4,5)-dimethylthiahiazo (-z-y1)-3,5-di- phenytetrazoliumromide (MTT)

Cell viability was measured using MTT Assay. Summarily cells were seeded in a 96-well plate. After reaching confluence, cells were stimulated with 0.1, 0.2, 1, 2, 10, 20, and 100 μM Rotigotine for 24 hours, succeeding that 15 µl of MTT assay labeling solution (Sigma-Aldrich, USA) was added into the plates, and they were then further incubated for 4 hours at 37°C. After the 4 hr incubation period, the medium was removed, 150 µl of dimethyl sulfoxide (DMSO) reagent was added, and absorbance was determined at 570 nm on a microplate reader.

### 2.3. Real-time PCR

After stimulation, HUVECs were harvested, and total RNA was isolated using an RNeasy Mini Kit (Qiagen, Germany). RT-PCR was used to synthesize cDNA from isolated RNA. The interpretation of the mRNA expression was then analyzed using cDNA and SYBR Green Master Mix (Bio-Rad, USA). The articulation levels of the target genes were determined by normalizing to the housekeeping standard (GAPDH) with the 2^−ΔΔCt^ technique. The following primers were used: SREBP-2, F: 5’-CCCTGGGAGACATCGACGA-3’, R: 5’-CGTTGCACTGAAGGGTCCA-3’; PCSK-9, F: 5’-CCTGCGCGTGCTCAACT-3’, R: 5’-GCTGGCTTTTCCGAAACTCT-3’; LDL-R, F: 5’-GTGTCACAGCGGCG-3’, R: 5’- CGCACTCTTTGATG-3’; NOX-4, F: 5’-TGTTGGATGACTGGAAACCA-3’, R: 5’-TGGGTCCACAACAGAAAACA-3’; TNF-α, F: 5’-TGTAGCCCATGTTGTAGCAAA-3’, R: 5’-CAAAGTAGACCTGCCCAGACT-3’; IL-8, F: 5’-ACTGAGAGTGATTGAGAGTGGAC-3’, R: 5’-AACCCTCTGCACCCAGTTT TC-3’; MCP-1, F: 5’-TTCTGTGCCTGCTGCTCATA-3’, R: 5’-CAGATCTCCTTGGCCACAAT-3’; ICAM-1, F: 5’-GGCCGGCCAGCTTATACAC-3’, R: 5’-TAGACACTTGAGCTCGGGCA-3’; VCAM-1, F: 5’-TCAGATTGGAGACTCAGTCATGT-3’, R: 5’-ACTCCTCACCTTCCCGCTC-3’.

### 2.4. Measurement of total cholesterol and free cholesterol

Quantitation of total cholesterol and free cholesterol was done after stimulation using Cholesterol Quantitation Kit (Sigma-Aldrich, USA) as per the manufacturer’s instructions.

### 2.5. Dihydroethidium (DHE) staining

After treatment, cells were then loaded with DHE (Sigma-Aldrich, USA) and kept at 37°C for 15 minutes. With a fluorescence microscope, the intracellular levels of reactive oxygen species (ROS) were assessed, and then the levels of intracellular ROS were measured using the Image J program.

### 2.6. Attachment of monocytes to HUVECs

HUVECs were incubated with 100 μg/mL ox-LDL with the inclusion or exclusion of 1 and 2 μM Rotigotine for 24 hours, followed by incubation with CMFDA-labeled U937 monocytes for 30 minutes at 37°C. The number of adherent U937 cells per visible field was determined by recording a video using the Leica DMS300 digital microscope (Leica, Shanghai), Images were recorded, and the attached cells were quantified from 10 frames in the off-line analysis using Metamorph automation software (Molecular Devices, USA).

### 2.7. Western blot analysis

HUVECs were lysed using cell lysis buffer and separated via 10% sodium dodecyl sulfate-polyacrylamide gel electrophoresis (SDS-PAGE) and transferred to polyvinylidene fluoride (PVDF) membranes (Bio-Rad, USA) for examination after stimulation [30]. The membranes were impeded with 5% nonfat dry milk before probing one night with primary antibodies and HRP-conjugated anti-IgG at 4°C. The blots were identified using an efficient chemiluminescence (ECL) kit (Sigma-Aldrich, USA). Western blot bands were digitized and kept. The software Image J was used to measure the levels of expression of target proteins. Target bands were carefully chosen for the film. Followed by subtraction of background and calculation of signal intensities, and data were exported for statistical analysis.

### 2.8. Enzyme-linked immunosorbent assay (ELISA)

The cell culture was collected, and levels of TNF-α (#SRP3177), IL-8 (#SRP3311), MCP-1 (#SRP3109), ICAM-1(#SRP6491), and VCAM-1(#RAB0505) were measured using correlating ELISA kits (Sigma-Aldrich, USA). Briefly, the standards were diluted to 5 gradient concentrations and were added to a 96-well plate along with the supernatants collected from each cultural medium. After incubation for 30 minutes at 37°C, the medium was removed, and the wells were washed using the washing solution. Subsequently, the wells were added with conjugate reagents followed by incubation for 30 minutes at 37°C. After washing, the TMB solution was added for coloration at 37°C for 15 minutes, followed by adding the stop solution to terminate the reaction. Lastly, the absorbance at 450 nm was measured using the microplate reader (Mindray, Shenzhen, China) [31].

### 2.9. Luciferase activity of NF-κB reporter

HUVECs were transfected with luciferase reporter plasmid pNF-κB-Luc and pRL-TK plasmid using LipofectAMINE 2000 (Invitrogen, USA) as per the manufacturer’s procedure to evaluate NF-κB transcriptional activity. PRL-SV40 Renilla luciferase control reporter vector was co-transfected as an internal control. The cells were treated with ox-LDL (100 μg/mL) in the inclusion or exclusion 1 and 2 M Rotigotine for 6 hours after transfection, for a total of 24 hours. a Dual-Luciferase Reporter Assay System (Promega, USA) was used to examine firefly luciferase activity. The data were presented as a fold change reference to non-treated cells.

### 2.10. Statistical analysis

Data were analyzed using the GraphPad Prism 9 software and presented as the Mean ±standard deviation (S.D.). Statistical analyses were performed using an analysis of variance (ANOVA) test. Bonferroni post-hoc test was done to access statistical differences between groups. The normality test was controlled by Kolmogorov-Smirnov test. The results supported the normality of our variables. P ˂ 0.050 was considered statistically significant.

## 3. Results

Using an ox-LDL- challenged HUVECs model, we investigated the effects of Rotigotine on AS. We tested the expressions of PCSK-9, SREBP-2, and LDL-R to measure the benefits of Rotigotine in total and free cholesterol deposition. Furthermore, we tested the effects of Rotigotine on oxidative stress and the expression of pro-inflammatory cytokines. In order to clarify whether Rotigotine inhibited the attachment of monocytes to HUVECs, we also tested the effect of Rotigotine on the expression of adhesion molecules. Lastly, we investigated the effect of Rotigotine on the activation of the NF-κB signaling pathway.

### 3.1. Cytotoxicity of Rotigotine in HUVECs

To determine the proper concentrations of Rotigotine to use in the subsequent experiments, we first tested for its cytotoxicity in HUVECs. The cells were stimulated with 0.1, 0.2, 1, 2, 10, 20, and 100 μM Rotigotine for 24 hours, and thereafter their viability was measured. As shown in Figure 1(b), the smaller concentrations of Rotigotine (0.1, 0.2, 1, and 2 μM) had no significant effect on the cell viability of the HUVECs. However, the higher concentrations (10, 20, and 100 μM) progressively decreased the cell viability, which resulted in the use of 1 and 2 μM Rotigotine for all subsequent experiments.

### 3.2. Treatment with Rotigotine reduced PCSK-9, SREBP-2, and LDL-R

**Figure 2.**
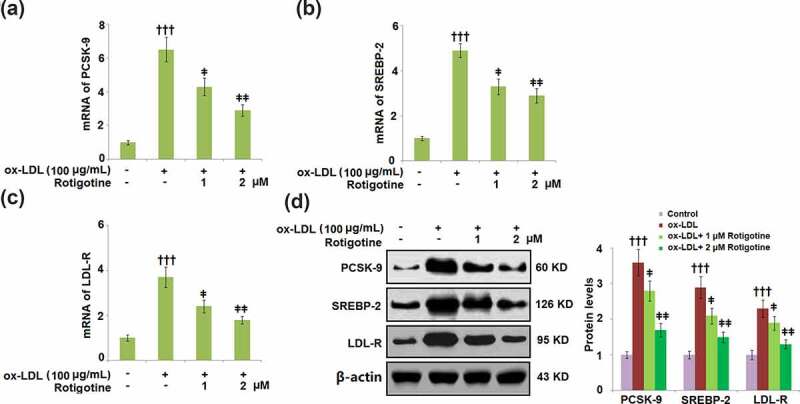
Treatment with Rotigotine reduced ox-LDL-induced expressions of PCSK-9, SREBP-2, and LDL-R in HUVECs. Cells were stimulated with ox-LDL (100 μg/mL) in the inclusion or exclusion of 1 and 2 μM Rotigotine for 24 hours. (a). mRNA of PCSK-9; (b). mRNA of SREBP-2; (c). mRNA of LDL-R; (d). Protein of PCSK-9, SREBP-2, LDL-R (†††, P < 0.0001 vs. vehicle group; ǂ, ǂǂ, P < 0.01, 0.001 vs. ox-LDL group, N = 6)

Figure 2(a-c) show that compared to the control, ox-LDL alone increased the mRNA expressions of PCSK-9, SREBP-2, and LDL-R. However, the introduction of 1 and 2 μM Rotigotine dose-responsively reduced these levels. Consistently, results in Figure 2(d) demonstrate that treatment with Rotigotine decreased the expression of PCSK-9, SREBP-2, and LDL-R at the protein levels.

### 3.3. Rotigotine decreases ox-LDL-induced total cholesterol and free cholesterol deposition in HUVECs

**Figure 3.**
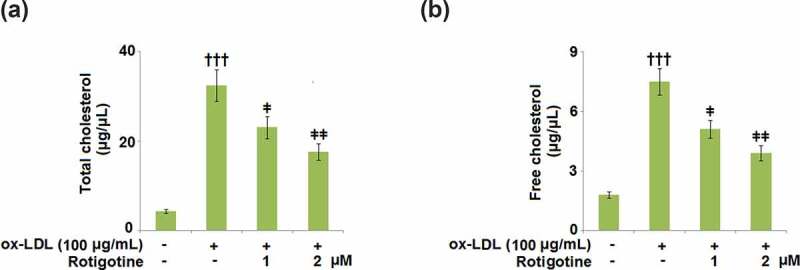
Rotigotine decreases ox-LDL-induced total cholesterol and free cholesterol deposition in HUVECs. Cells were stimulated with ox-LDL (100 μg/mL) in the inclusion or exclusion of 1, and 2 μM Rotigotine for 24 hours. (a). Total cholesterol (TC); (b). Free cholesterol (†††, P < 0.0001 vs. vehicle group; ǂ, ǂǂ, P < 0.01, 0.001 vs. ox-LDL group, N = 5–6)

Next, we checked whether or not Rotigotine ameliorated the ox-LDL-induced increase in cholesterol in the HUVECs. We found that although ox-LDL increased the total cholesterol from 4.3 ± 0.41 to 32.5 ± 3.52 μg/μL, treatment with 1 and 2 μM Rotigotine reduced it to 23.1 ± 2.45 and 17.6 ± 1.86 μg/μL, respectively (Figure 3(a)). Likewise, the free cholesterol level was increased from 1.8 ± 0.16 to 7.5 ± 0.68 μg/μL by ox-LDL, but the introduction of the two doses of Rotigotine decreased it to 5.1 ± 0.45 and 3.9 ± 0.38 μg/μL, respectively (Figure 3(b)).

### 3.4. Treatment with Rotigotine attenuated ox-LDL- induced oxidative stress in HUVECs

**Figure 4.**
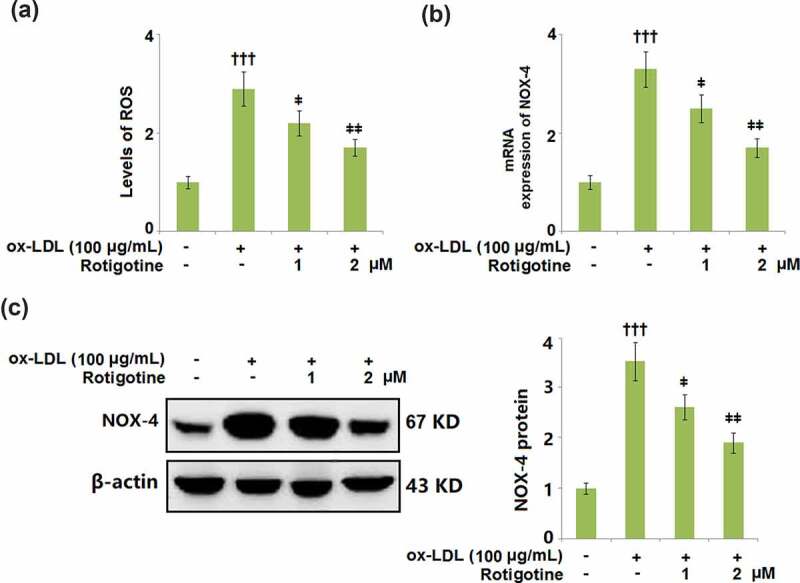
Treatment with Rotigotine attenuated ox-LDL-induced oxidative stress in HUVECs. (a). Levels of ROS were measured using DHE staining; (b). m RNA expression of NOX-4; (c). Protein of NOX-4 (†††, P < 0.0001 vs. vehicle group; ǂ, ǂǂ, P < 0.01, 0.001 vs. ox-LDL group, N = 5–6)

We then measured the levels of the oxidative stress markers ROS and NOX-4 to determine whether Rotigotine had an effect on the oxidative stress induced by ox-LDL. Figure 4(a) shows the levels of ROS and their increase due to stimulation with ox-LDL. Following treatment with Rotigotine, the ROS levels were dose-responsively decreased. Similarly, ox-LDL increased the mRNA and protein expression of NOX-4, however, it was dose-responsively reduced by the two doses of Rotigotine (Figure 4(b,c)).

### 3.5. Rotigotine inhibited the expression of pro-inflammatory cytokines in HUVECs

**Figure 5.**
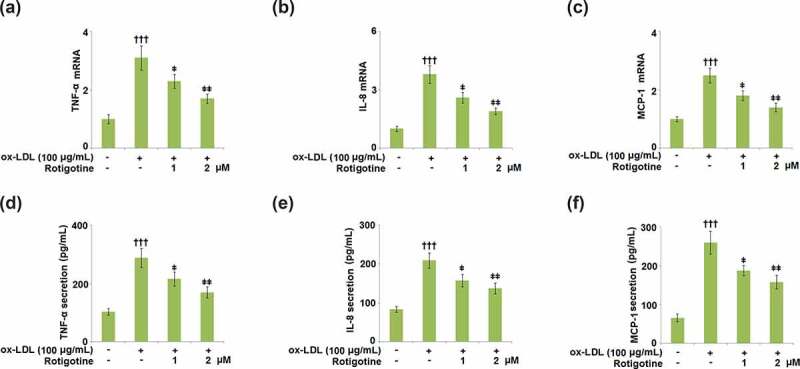
Rotigotine inhibited the expression of pro-inflammatory cytokines in HUVECs. (a). TNF-α mRNA; (b). IL-8 mRNA; (c). MCP-1 mRNA; (d). TNF-α secretion as measured with ELISA; (e). IL-8 secretion as measured with ELISA; (f). MCP-1 secretion as measured with ELISA (†††, P < 0.0001 vs. vehicle group; ǂ, ǂǂ, P < 0.01, 0.001 vs. ox-LDL group, N = 5–6)

The mRNA and secretions of the pro-inflammatory cytokines TNF-α, MCP-1, and IL-8 were measured to determine the effect of Rotigotine. Figure 5(a-c) show that ox-LDL increased the mRNA levels of the pro-inflammatory cytokines. However, treatment with 1, and 2 μM Rotigotine ameliorated these increased levels dose-responsively. The secretion of TNF-α was significantly increased by ox-LDL from 102.5 ± 11.3 pg/mL to 288.6 ± 32.5 pg/mL, with the introduction of the two doses of Rotigotine reducing it to 215.5 ± 23.8 pg/mL and 169.8 ± 18.5 pg/mL, respectively (Figure 5(d)). Ox-LDL also increased the secretion of IL-8 from 83.1 ± 7.2 pg/mL to 209.1 ± 19.6 pg/mL, which was later reduced to 157.8 ± 16.2 pg/mL and 137.6 ± 13.4 pg/mL by 1 and 2 μM Rotigotine, respectively (Figure 5(e)). Similarly, Ox-LDL elevated the secretion of MCP-1 from 65.3 ± 9.9 to 259.1 ± 29.6 pg/mL, which was reduced to 187.8 ± 13.2 and 157.6 ± 17.4 pg/mL by 1 and 2 μM Rotigotine (Figure 5(f)).

### 3.6. Rotigotine suppressed the expression of cell adhesion molecules in HUVECs

**Figure 6.**
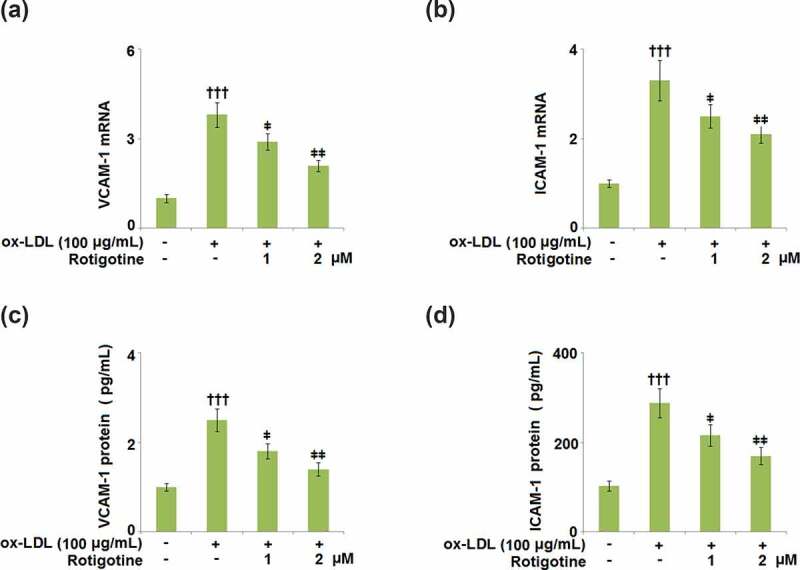
Rotigotine suppressed the expression of cell adhesion molecules in HUVECs. (a). VCAM-1 mRNA; (b). ICAM-1 mRNA; (c). VCAM-1 protein as measured with ELISA; (d). ICAM-1 protein as measured by ELISA (†††, P < 0.0001 vs. vehicle group; ǂ, ǂǂ, P < 0.01, 0.001 vs. ox-LDL group, N = 5–6)

VCAM-1 and ICAM-1 cause leukocyte adhesion to the endothelium and are involved in all stages of AS. They have been shown to be increased in cholesterol-rich diets, therefore, we examined the effects of Rotigotine on their ox-LDL-induced increased expression. The mRNA levels of both VCAM-1 (Figure 6(a)) and ICAM-1 (Figure 6(b)) were increased by ox-LDL, but dose-responsively reduced by 1, and 2 μM Rotigotine. The protein level of VCAM-1 was increased from 136.6 ± 14.2 to 387.8 ± 45.7 pg/mL by ox-LDL, but the two doses of Rotigotine reduced it to 285.4 ± 31.6 and 233.7 ± 21.5 pg/mL, respectively (Figure 6(c)). Similarly, Figure 6(d) shows that ox-LDL notably increased the protein level of ICAM-1 from 95.5 ± 9.2 to 296.6 ± 26.8 pg/mL. The introduction of 1, and 2 μM Rotigotine decreased the protein level of ICAM-1 to 216.3 ± 19.1 and 162.5 ± 17.2 pg/mL, respectively.

### 3.7. Rotigotine ameliorated ox-LDL- induced attachment of monocytes to HUVECs

**Figure 7.**
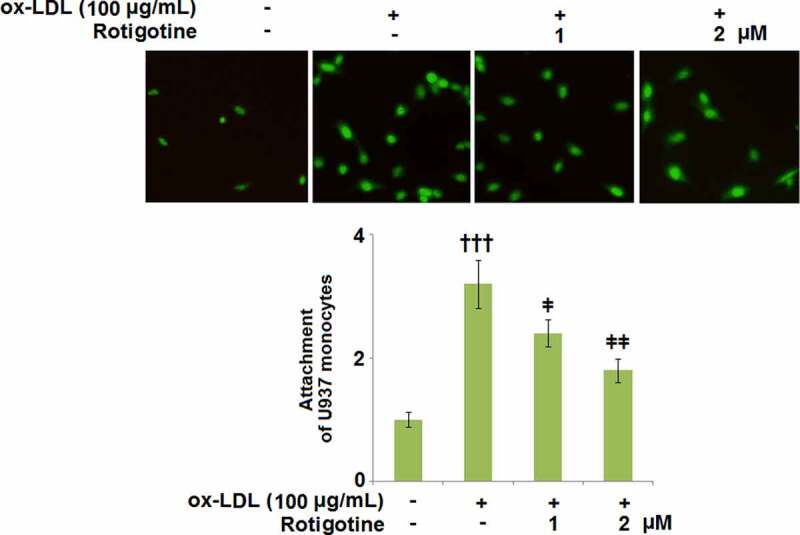
Rotigotine ameliorated ox-LDL- induced attachment of monocytes to HUVECs. Attachment of U937 monocytes to HUVECs was measured using 5-chloromethylfluorescein diacetate (CMFDA) staining (†††, P < 0.0001 vs. vehicle group; ǂ, ǂǂ, P < 0.01, 0.001 vs. ox-LDL group, N = 5–6)

Figure 7 shows that following stimulation with ox-LDL, U937 monocytes attached more to the HUVECs. However, when the two doses of Rotigotine were introduced, the monocytes exhibited a considerably decreased attachment to the HUVECs.

### 3.8. Rotigotine inhibited activation of NF-κB

**Figure 8.**
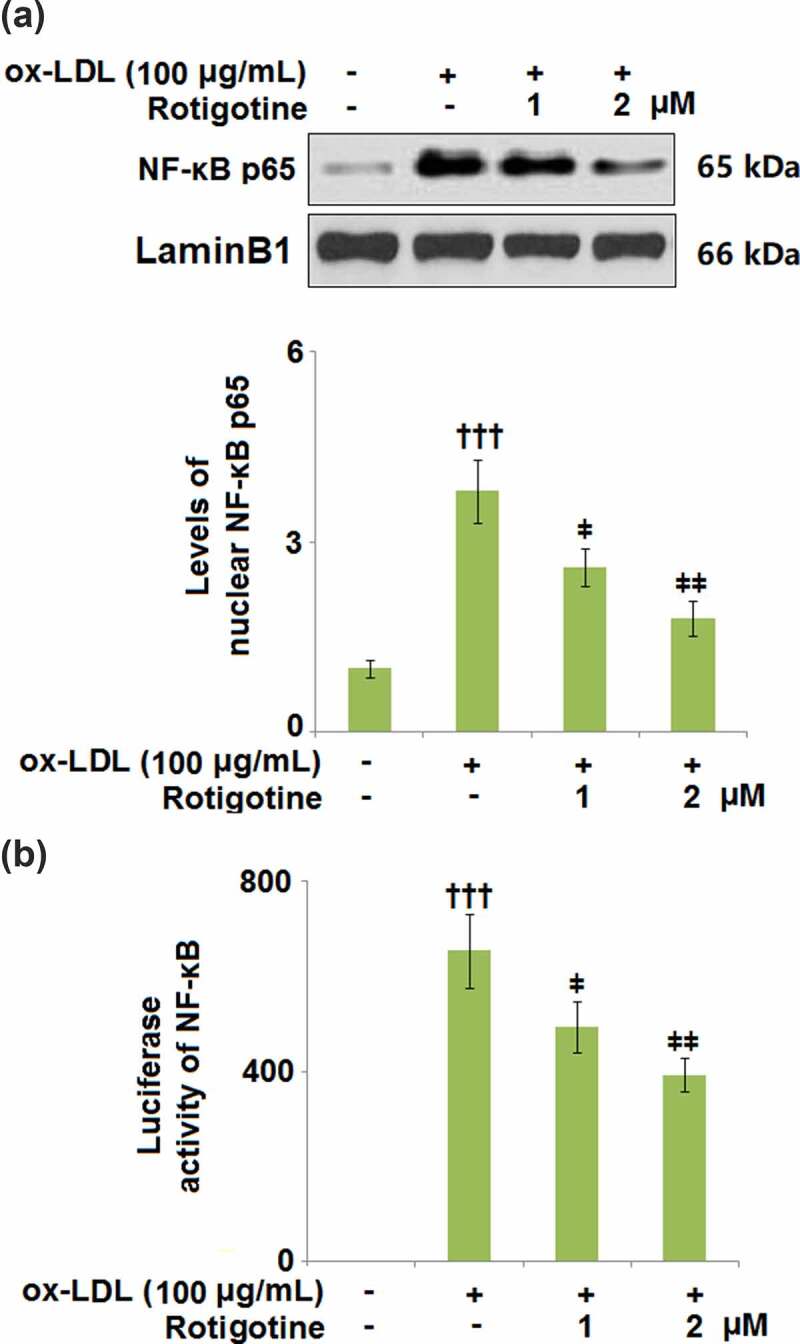
Rotigotine inhibited activation of NF-κB. (a). Levels of nuclear NF-κB p65; (b). Luciferase activity of NF-κB (†††, P < 0.0001 vs. vehicle group; ǂ, ǂǂ, P < 0.01, 0.001 vs. ox-LDL group, N = 5–6)

NF-κB plays an important role in inducing inflammatory genes involved in response to injury and infection. To clarify the involvement of NF-κB, we used SC75741, an NF-κB inhibitor, to inhibit its activity. The results in Supplementary Figures 1A and 1B show that inhibition of NF-κB reduced the expressions of VCAM-1 and ICAM-1 against ox-LDL. Furthermore, the attachment of monocytes was also suppressed by the presence of SC75741 (Supplementary Figure 1 C). Here, we found that ox-LDL increased the levels of nuclear NF-κB p65, which were later reduced by 1 and 2 μM Rotigotine, respectively (Figure 8(a). Figure 8(b) shows that the luciferase activity of NF-κB was also increased by ox-LDL but dose-responsively reduced by the two doses of Rotigotine.

## 4. Discussion

Aided by EC dysfunction, AS is considered a chronic inflammatory ailment [32]. PCSK-9 decreases LDL intake by enhancing LDL-R degradation and preventing its recirculation to the cell surface [13]. AS can result from familial hypercholesterolemia that is established following genetic defects in PCSK-9 [33,34]. SREBP-2 controls the gene transcriptions of PCSK-9 and LDL-R, which are co-expressed in various cells [35–37]. In this study, we observed a reduction in PCSK-9 in the HUVECs after Rotigotine supplementation. Consistent with previous investigations [38], we found that ox-LDL induces the expression of PCSK-9, implicating an inflammatory response in its activation. Also shown is the inhibition of SREBP-2, which regulates both PCSK-9 and LDL-R genes, by Rotigotine. Therefore, we concluded that the protective effects of Rotigotine against AS are partly exerted by restricting the expressions of LDL-R, PCSK-9, and SREBP-2. Under atherosclerotic conditions, ox-LDL is engulfed by macrophages, resulting in the accumulation of cellular cholesterol. Recent studies have shown that there is a correlation between atherosclerosis-associated inflammation and the accumulation of free cholesterol in macrophages, which leads to NLRP3 inflammasome activation [39]. Our results demonstrate a complete correlation to this, as we found elevated free and total cholesterol in the HUVECs induced by ox-LDL. However, supplementation with Rotigotine mitigated this cholesterol increase. Alongside ox-LDL-induced ROS generation, another well-established AS development risk is the generation of ROS by NOX-4 [40,41]. ECs are some of the cells that exhibit a high expression of NOX-4 [42], and in our study, we reported upregulated NOX-4 and ROS expressions in HUVECs following incubation with ox-LDL. Rotigotine ameliorated the increased ROS and NOX-4 expressions in the HUVECs. From these results, we can infer that Rotigotine’s ability to suppress NOX-4 mRNA may be the reason for the reduced ROS levels, which is consistent with a previous report [43].

MCP-1, ICAM-1, and VCAM-1 are some of the adhesion proteins induced by the activation of ECs, and when macrophages engulf ox-LDL, it results in their transformation into foam cells. The resulting endothelial dysfunction and atherosclerotic plaque formation come from these foam cells triggering inflammatory cascades [44–47]. Our results have shown that treatment with Rotigotine attenuated the ox-LDL-induced overexpression of ICAM-1 and VCAM-1 on the HUVECs. Furthermore, the expressions of MCP-1, IL-8, and TNF-α were also kept in check by Rotigotine. Ox-LDL has been shown to initiate the aggregation of monocytes and U937 cells. The same study also showed that ox-LDL stimulated the adhesion of U937 cells to ECs, indicating that it contributes to the progression of atherosclerosis in part by inducing the adhesion of U937 monocytes to the vessel walls [24]. Similarly, we reported an extensive attachment of U937 monocytes to the HUVECs induced by their incubation with ox-LDL. The introduction of Rotigotine, however, had an inhibitory effect on this attachment of the U937 monocytes. In mononuclear phagocytes, ox-LDL alters the expression of inflammatory gene products.

The activation of NF-κB, a transcription factor important in controlling the expressions of these genes, was examined to highlight the mechanisms involved in this effect of ox-LDL. When the macrophages were pretreated with ox-LDL, NF-κB was activated in response to either the combination of interferon-gamma (IFN-gamma) and IL-2 or lipopolysaccharide (LPS). The degree of LDL oxidation was also shown to be directly proportional to the effects of ox-LDL on NF-κB activation [48]. The presence of activated NF-κB in human atherosclerotic lesions implies its involvement in AS, and when human THP-1 monocytes were incubated with ox-LDL, NF-κB p65 was activated, and the expressions of pro-inflammatory cytokines induced [25]. Consistently, our results showed increased nuclear NF-κB p65 levels and NF-κB luciferase activity in the HUVECs incubated with ox-LDL, whereas the supplementation with Rotigotine reversed these effects, indicating its inhibition of the activation of NF-κB. However, there have been conflicting reports on the effect of ox-LDL treatment on the activation of NF-κB in ECs. Some authors have reported enhanced NF-κB activity [26,27], while others found the activation of NF-κB to be suppressed [49].

It has been reported that dopamine receptors are expressed in endothelial cells and play an important role in regulating the physiological function of vascular endothelial cells [50,51]. In this study, we found that Rotigotine, the nonergolinic dopamine agonist, exerted therapeutic benefits in the ox-LDL-challenged HUVECs model. However, there are still some limitations. It is still unknown whether the beneficial effects of Rotigotine are independent or dependent on dopamine receptors. Additionally, it is well known that Rotigotine could activate all five types of dopamine receptors. The subtype of dopamine receptors that could mediate the pharmacological function in endothelial cells needs to be elucidated.

## 5. Conclusion

Taken together, our findings show that Rotigotine suppressed the expressions of PCSK-9, SREBP-2, and LDL-R. It also prevented ox-LDL-induced cholesterol accumulation in ECs, inhibited the activation of NF-κB, ameliorated the attachment of U937 monocytes, and decreased the expression of NOX-4 and generation of ROS in ECs. Furthermore, Rotigotine inhibited the expressions of ICAM-1 and VCAM-1 in HUVECs and displayed anti-inflammatory potential in ox-LDL-challenged HUVECs. These findings suggest that the effects of Rotigotine on HUVECs serve to suppress the oxidative stress and pro-inflammatory cascades in the atherosclerotic plaques and could, therefore, potentially have a therapeutic role in AS.

## Supplementary Material

Supplemental Material

## Data Availability

Data of this study are available upon reasonable request to the corresponding authors.
