# Supplementary material for: Rotigotine protects against oxidized low-density lipoprotein(ox-LDL)-induced damages in human umbilical vein endothelial cells(HUVECs)
Source: Bioengineered. 2021 Dec 3;12(2):10568–79. doi: 10.1080/21655979.2021.2000224 (PMC8810014; doi:10.1080/21655979.2021.2000224)
Supplement: Supplemental Material [file KBIE_A_2000224_SM6675.zip › supplementary/Supplementary Figure legend.docx]

**Supplementary Figure 1.** Inhibition of NF-κBwith its inhibitor SC75741 suppressed attachment of U937 monocytes. Cells were stimulated with ox-LDL (100 μg/mL) in the presence or absence of SC75741 (5 μM) for 24 hours. (A). VCAM-1 mRNA; (B). ICAM-1 mRNA; (C). Attachment of U937 monocytes (†††, P<0.0001 vs. vehicle group; ǂǂ, P<0.01, 0.001 vs. ox-LDL group, N=6).
