# Supplementary figures and images for: Rotigotine protects against oxidized low-density lipoprotein(ox-LDL)-induced damages in human umbilical vein endothelial cells(HUVECs)
Source: Bioengineered. 2021 Dec 3;12(2):10568–79. doi: 10.1080/21655979.2021.2000224 (PMC8810014; doi:10.1080/21655979.2021.2000224)

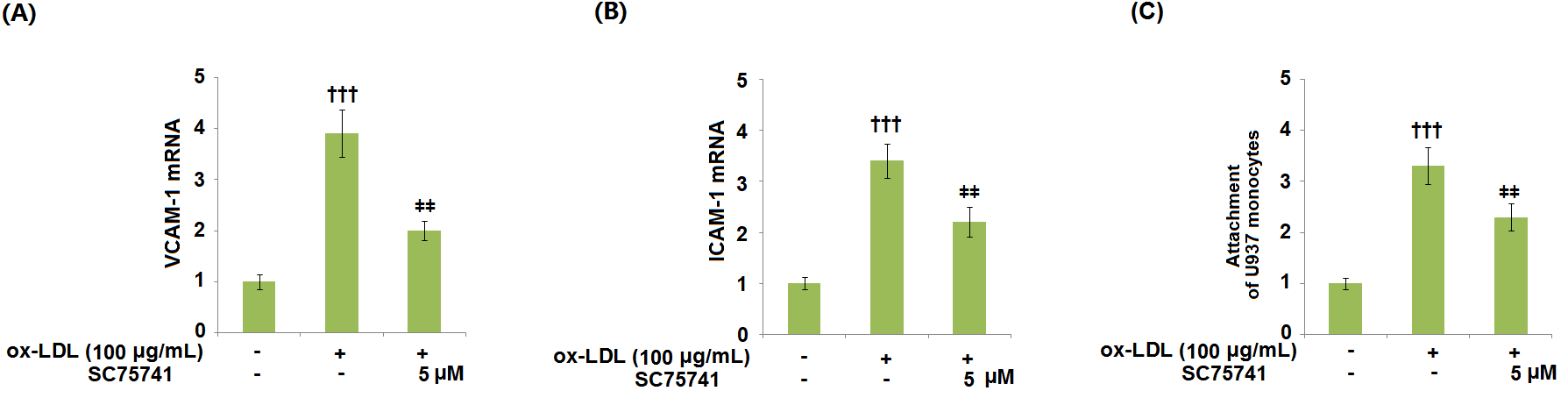

Supplement: Supplemental Material [file KBIE_A_2000224_SM6675.zip › supplementary/supplementary_figure1.tif]
